# Improved Response of ZnO Films for Pyroelectric Devices

**DOI:** 10.3390/s121217007

**Published:** 2012-12-12

**Authors:** Chun-Ching Hsiao, Shih-Yuan Yu

**Affiliations:** Department of Mechanical Design Engineering, National Formosa University, No. 64, Wunhua Rd., Huwei Township, Yunlin County 632, Taiwan; E-Mail: Shih.Yuan.Y@gmail.com

**Keywords:** zinc oxide, aerosol deposition, electrode, pyroelectricity

## Abstract

Increasing the temperature variation rate is a useful method for enhancing the response of pyroelectric devices. A three-dimensional ZnO film was fabricated by the aerosol deposition (AD) rapid process using the shadow mask method, which induces lateral temperature gradients on the sidewalls of the responsive element, thereby increasing the temperature variation rate. To enhance the quality of the film and reduce the concentration of defects, the film was further treated by laser annealing, and the integration of a comb-like top electrode enhanced the voltage response and reduced the response time of the resulting ZnO pyroelectric devices.

## 1. Introduction

Zinc oxide (ZnO) is a distinctive material because it possesses such properties as semiconductivity (II–VI compound semiconductors), piezoelectricity and pyroelectricity without the poling process, and it also has a low cost, low toxicity and is environmentally friendly. Interesting properties, including a large band gap (∼3.3 eV), high transmittance, excellent thermal stability and n or p conductivity, are other attributes of ZnO material. Wide band gap wurtzite phase ZnO has attracted attention due to its versatility in many applications, such as blue and ultraviolet light emitters, transparent conductors, solar cell windows, gas sensors, photovoltaic devices, pyroelectric sensors, surface acoustic wave (SAW) devices, film bulk acoustic resonators (FBARs), catalysis, optoelectronics and photo-electrochemical devices [1–3].

ZnO films have been synthesized by numerous methods, such as metal organic chemical vapor deposition, molecular beam epitaxy, magnetron sputtering, pulsed laser deposition, atomic layer deposition, spray pyrolysis, the filtered cathodic vacuum arc technique and the sol-gel process. The quality of ZnO films obtained by the above methods depends on the specific growth methods and conditions. Thus, the preferential orientation of ZnO films depends on the growth conditions. The most densely packed and thermodynamically favorable growth orientation in the ZnO wurtzite structure is one in which the c-axis is perpendicular to the substrate. ZnO films with the c-axis normal to the substrate are preferred in many applications, such as pyroelectric devices [4,5] and film bulk acoustic resonators [6].

The pyroelectricity of ZnO is attributable to non-centrosymmetrical crystals, and so it has a specific polar axis along the direction of spontaneous polarization [4,5]. When ZnO is subjected to temperature variations, its internal polarization will produce an electric field. Pyroelectric materials respond to changes in temperature, which cause internal strain and in turn result in an electrical charge on the material surface. Therefore, increasing the response of a ZnO pyroelectric sensor depends on increasing the temperature variation rate of the ZnO layer, adopting a ZnO film with a strongly preferred orientation towards the C-axis and using a high-performance thermal-isolation structure. Moreover, a partially covered top electrode has been proven to result in a higher response than a fully covered electrode, because it allows the pyroelectric layer to make direct contact with the heat source, thus increasing the temperature variation rate [4]. To enhance the temperature variation rate in pyroelectric films, a three-dimensional pattern is etched on the responsive element of LiTaO_3_ with lateral temperature gradients induced on the sidewalls of the responsive element under homogeneous irradiation for improving the response of pyroelectric devices [7]. Recently, pyroelectric energy modules offer novel energy conversion technology by transforming waste heat into electricity, which converts the time-dependent temperature variations into electrical energy. Pyroelectric energy conversion devices require the thermal cycling of a pyroelectric element between a hot and a cold temperature source to produce electricity [8–11]. Using the concept of lateral temperature gradients induced on the sidewalls of the responsive element, etching the PZT material to produce deeper cavities and a smaller electrode width can effectively enhance the temperature variation rate in a thicker PZT material, but the PZT etchant with a low etching rate is unsuitable to dig the deeper cavities in the PZT sheet [12]. Furthermore, while trenching the PZT sheets to produce deeper trenches can effectively enhance the temperature variation rate in a thicker PZT pyroelectric cell as a result of the lateral temperature gradients induced by the trenched electrode [13]. Hence, a three-dimensional pattern on pyroelectric elements or layers is positive for ameliorating the temperature variation rate and enhancing the response of pyroelectric devices. Besides, the Olsen cycle enables the generation of significant electrical energy using the pyroelectric effect. The Olsen cycle consists of two isothermal and two isoelectric field processes in the displacement *versus* the electric field diagram. A dipping experiment is used to perform the Olsen cycle by a piston oscillating vertically and driving silicon oil back and forth between a heat source and a cold heat exchanger in a Teflon cylindrical chamber [10]. Moreover, a stamping experiment is also used to implement the Olsen cycle by alternatively placing a pyroelectric material for heat conduction with a cold and a hot source [8]. Therefore, the various heat transfers also affect the efficiency of the Olsen cycle.

Aerosol deposition (AD) is a novel deposition method developed by Akedo *et al.* In this technique, ceramic films are prepared by ejecting an aerosol consisting of a mixture of ultra-fine ceramic particles and gas from the nozzle to the substrate without vaporization of materials. AD has become very attractive for the deposition of ceramic films at room temperature on several kinds of substrates, such as silicon, glass, plastic and metal. In AD, individual sub-micrometer particles of the materials being deposited are mixed with a carrier gas in the aerosol chamber to generate an aerosol flow. The flow is ejected through a micro-orifice nozzle and deposited onto a substrate in the deposition chamber, which is vacuumed during the deposition. Particle velocities are controlled by the gas flow consumption and measured with a mass flow controller. Compared with the sputtering, screen-printing or sol-gel methods, AD provides many advantages for producing films in the range of 1∼100 μm thickness with a high deposition rate, low deposition temperature and low cost [14]. The AD method can achieve fine results with a three-dimensional patterning [15,16] and fabricate a dense structure by a reduction in crystallite size by fracture or plastic deformation at room temperature [14,17].

Although ZnO films grown at a low temperature are available from the AD process, an annealing treatment is necessary to further improve its quality and modulate its properties. Annealing treatment is one of the most common methods for reducing the defects and improving the quality of as-grown thin films [18,19]. Laser annealing of thin films is a technique of rapid crystallization that has the benefits of area selectivity, rapid local heating, no high temperature bulk heating, minimal disturbance to the surrounding area and flexible management [1,20]. Hence, laser annealing was adopted to improve the ZnO film quality in the present study, as it allowed for additional processing on devices that could not be processed at elevated temperatures. Moreover, in contrast with the thermal annealing treatment, laser irradiation can effectively reduce the residual stress and grain size in ZnO film [20].

In the present study, the preparation of ZnO films by AD was investigated. Moreover, a three-dimensional ZnO film fabricated by an AD rapid process with the shadow mask method which induces lateral temperature gradients on the sidewalls of the responsive element, thereby increasing the temperature variation rate and improving the response of pyroelectric sensors was applied to fabricate pyroelectric devices. Laser annealing was used to further improve the ZnO film quality. Then, the crystalline phases and the morphology of the ZnO films were characterized by X-ray diffraction (XRD) and scanning electron microscopy (SEM), respectively.

## 2. Materials and Methods

### 2.1. Preparation of ZnO Films

**Figure 1.**
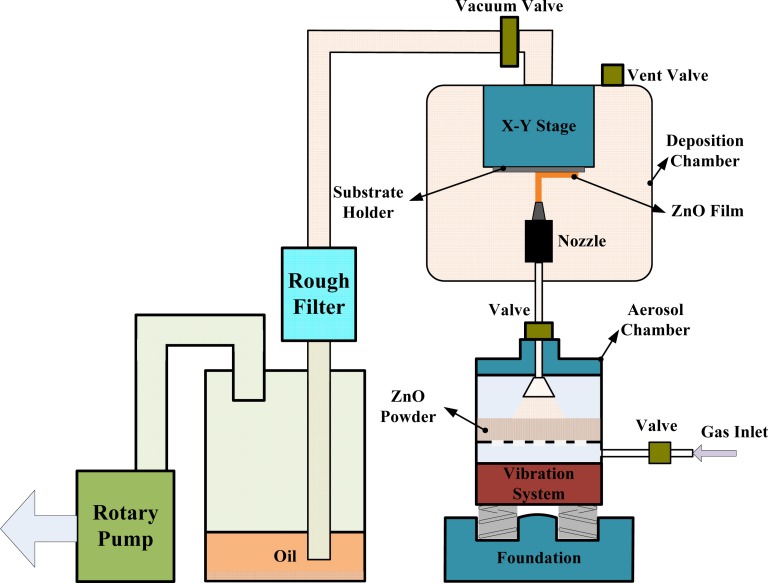
Apparatus for aerosol deposition system.

Figure 1 shows the schematic diagram of the AD apparatus, which consisted of both an aerosol and a deposition chamber. The aerosol chamber generated the ZnO aerosol. The aerosol flow of ZnO powder was formed by a carrier gas (nitrogen) and a vibration system. The vibration system was used to mix the ZnO powder with the carrier gas. The aerodynamic filter in the aerosol chamber was used to discard the agglomerated particles. The deposition chamber, used to form the films, consisted of an X–Y stage, a nozzle and a substrate holder with a heating system. This chamber was evacuated during the film deposition process by a rotary vacuum pump with a dust collection system. The fine ZnO powder flow in the aerosol chamber was delivered to the deposition chamber by a pressure difference between the aerosol and deposition chambers. The fine ZnO powder flowed through the nozzle, and then impacted and deposited on the substrate to form the ZnO film. Particle velocities were controlled by the gas flow consumption and measured with a mass flow controller.

The starting powder was commercially available ZnO (Top Nano Technology Co. Ltd., New Taipei, Taiwan). The properties of the starting ZnO powder are shown in Table 1. Table 2 shows the deposition parameters used for the AD method. Starting powder with high moisture content reduces film quality, as agglomerated particles act as a cushion to absorb the kinetic energy, which results in the formation of compacted powders when the powder impacts against the substrate. Therefore, the pretreatment of the ZnO starting powder was carried out to improve the ZnO film quality. The powder was subjected to heat treatment at 150 °C for 1 h using an oven. ZnO film with a thickness of 3 μm was deposited on the substrate, which consisted of 525 μm thick silicon and 1 μm thick silicon nitride, by AD at room temperature employing various process parameters. Then, laser annealing was applied to the ZnO films to enhance the film quality.

The laser annealing system (LEE-25, Laser Life, Taipei, Taiwan), using continuous-wave CO_2_ laser irradiation with a wavelength of 10.6 μm, power of 25 W and beam diameter of 2 mm, was adopted for the ZnO film annealing in a N_2_ atmosphere. The laser annealing parameters comprised the movement velocity of the laser head in the X–Y plane, distance between substrates and laser head, laser power adjustment, ambient gas and flow rate, as shown in Table 3.

The film thickness was measured by a surface analyzer (ET-4000AK, Kosaka, Tokyo, Japan). The crystalline phases of the ZnO films were identified by X-ray diffraction (XRD, PANalytical, X’Pert PRO MPD, Almelo, The Netherlands). The morphology was characterized by scanning electron microscopy (SEM).

### 2.2. Structure and Fabrication of ZnO Thin-Film Pyroelectric Devices

ZnO thin-film pyroelectric devices are comprised of a ZnO pyroelectric layer sandwiched between top and bottom electrodes and built on a thermal-isolation structure to block heat loss. The top side is exposed to a heat source. The principle of thin-film pyroelectric sensors is based on the pyroelectric effect, which converts the temperature variation to the corresponding electrical signal [4,5]. The dynamic pyroelectric response current of thin-film pyroelectric sensors can be described using Equation (1):

(1) $$i_{p}=\eta \times P\times A\times \mathrm{dT}/\mathrm{dt}$$

where *η* is the absorption coefficient of radiation, *P* is the pyroelectric coefficient of the pyroelectric film, *A* is the sensing area and *dT/dt* is the temperature variation rate of the pyroelectric film. The thermal-isolation structure, sensitive material properties, top-electrode layout and absorption coefficient are the most important performance-enhancing qualities of pyroelectric devices. From Equation (1), it can be seen that a higher temperature variation rate in pyroelectric films leads to a higher response current in the pyroelectric devices. Moreover, increasing the heat absorption of pyroelectric films can also improve the response of pyroelectric devices.

**Figure 2.**
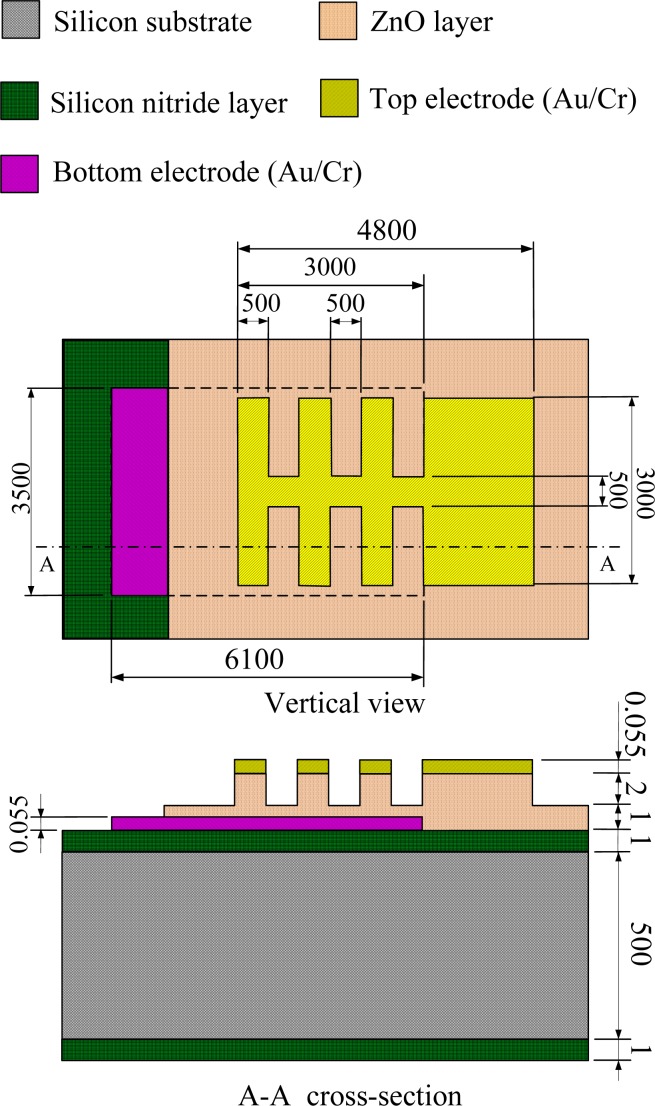
Schematic diagram of the multilayer ZnO pyroelectric device with the comb-like electrode and the three-dimensional ZnO film. (unit: μm).

Temperature variation rates in pyroelectric layers seriously affect the response of pyroelectric sensors. A partially covered top electrode has a higher response than a fully covered top electrode because the uncovered part of the ZnO layer is directly exposed to the heat source and, thus, markedly increases the heat absorption [5]. Besides, a three-dimensional pattern in pyroelectric materials induces lateral temperature gradients on the sidewalls of the responsive element for increasing the temperature variation rate [7]. In the present study, the AD method was used to deposit a three-dimensional ZnO film integrated with a comb-like partially covered top electrode to enhance the performance of ZnO pyroelectric devices. The schematic diagram and dimensions of the pyroelectric device with the three-dimensional ZnO film and the comb-like electrode are detailed in Figure 2.

**Figure 3.**
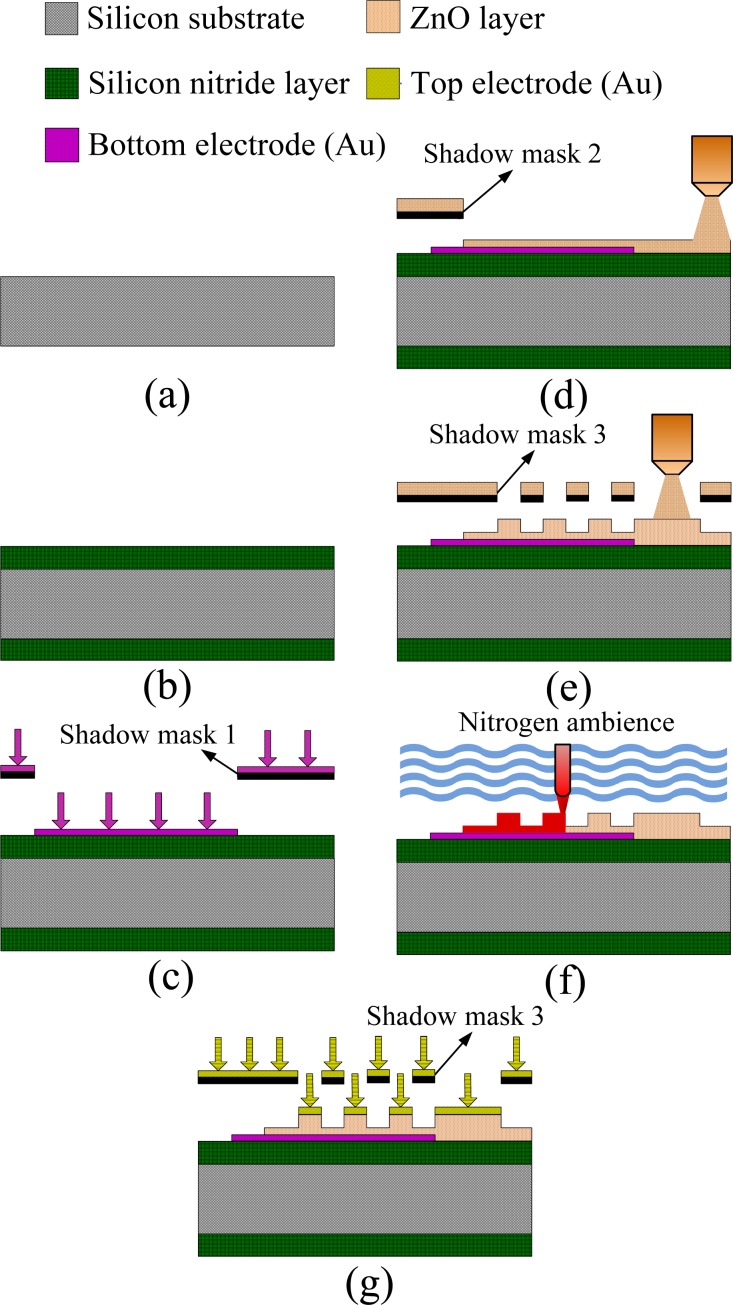
Process flow of the multilayer ZnO pyroelectric device with the comb-like electrode and the three-dimensional ZnO film.

The fabrication flow of the ZnO pyroelectric devices was divided into several steps as follows: a silicon wafer with both sides polished was used as a substrate to support the multilayer ZnO pyroelectric devices, as shown in Figure 3(a). A Si_3_N_4_ layer of 1 μm thickness was deposited on both sides of the silicon wafer by low-pressure chemical vapor deposition (LPCVD), as shown in Figure 3(b). The silicon nitride layer obstructs the thermal conduction to the silicon substrate and enhances the heat absorption in the pyroelectric layers. The bottom electrode was deposited on the substrates by electron beam evaporation and patterned by the shadow mask method, as shown in Figure 3(c). The bottom electrode was composed of gold and chromium. The latter was an adhesion layer to promote the adhesion between the gold electrode and the substrate. The thickness of the gold and chromium was 50 nm and 5 nm, respectively. The next step was to deposit ZnO films with a thickness of 1 μm by AD and pattern by a shadow mask to expose the bottom electrode, as shown in Figure 3(d). Then, ZnO film with a thickness of 2 μm was deposited on the preceding ZnO film and patterned by a shadow mask with a comb-like form, as shown in Figure 3(e). These ZnO films promoted by laser annealing ensued, as shown in Figure 3(f). The processing step in Figure 3(g) was used to deposit the top electrode on the ZnO layer. The composition and deposition of the top electrode were similar to those of the bottom electrode. The top electrode was also patterned by the shadow mask with a comb-like form. The shadow mask method simplified the processing steps, shortened the processing time and further avoided chemical corrosion to the ZnO films during the photolithography process.

### 2.3. Signal Measurement

**Figure 4.**
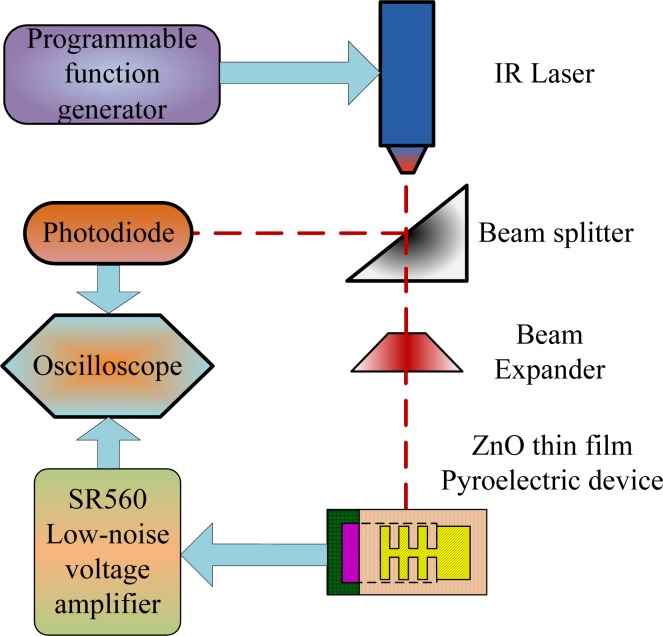
Schematic diagram of responsivity measurement system.

A response measurement system (Figure 4) was used to determine the output signals of the present ZnO pyroelectric devices. The radiation source was a calibrated infrared (IR) laser of 900 nm wavelength and 7 mW maximum power. The laser beam was chopped and molded as a square wave with a modulated frequency (ω) by a programmable function generator. A prism was used to split the modulated beam into two beams. The two beams had the same power: one was reflected on a photodiode as the reference signal, and the other was expanded via a beam expander, such that the beam spot covered the entire region of the patterned top electrode of the ZnO pyroelectric sensors. A reference signal produced by the photodiode was compared with the output signals generated by the present sensors on the oscilloscope. The output voltage of the sensor was treated using an SR560 low-noise voltage amplifier. Finally, a digital oscilloscope was used to record and display both the output signals of the sensors and the photodiode.

## 3. Results and Discussion

**Figure 5.**
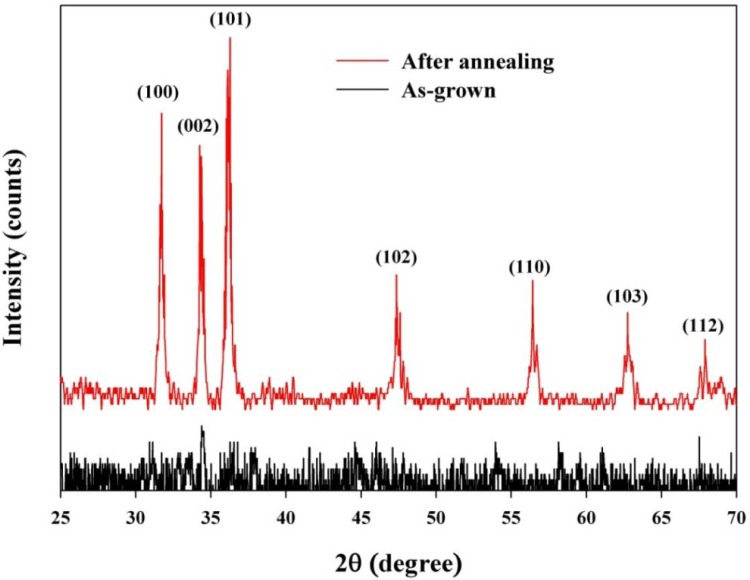
XRD patterns of the ZnO films with as-grown and laser annealing.

AD is a form of gas deposition method or jet printing method without vaporization of the material, which is a novel and very attractive coating method for ceramic integration. A high deposition rate and a low deposition temperature are the main advantages for AD, which can accelerate the fabrication of films applied on microdevices, sensors or actuators. AD is an excellent candidate for fabricating low cost and highly reliable embedded electric devices in printed circuit boards. Figure 5 shows the XRD patterns for the ZnO films with as-grown and laser annealing.

**Figure 6.**
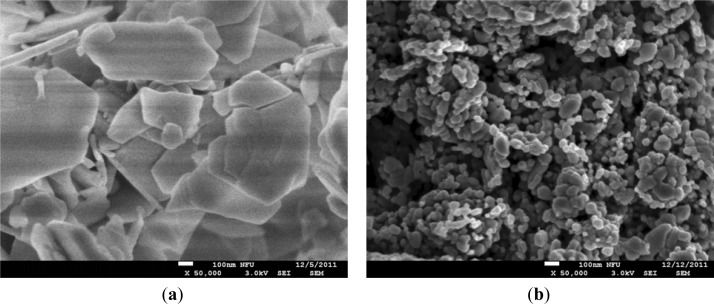
SEM micrographs of the ZnO films: (**a**) as-grown and (**b**) after laser annealing.

The laser-irradiated ZnO film exhibited a hexagonal wurtzite phase consistent with crystalline materials. Figure 6 shows the SEM micrographs for the ZnO films with as-grown and laser annealing. The as-grown ZnO films were observed to have sheet-like grains, which were consistent with the starting particles of the ZnO powder. Laser annealing is a rapid and local heating and cooling process, which can also result in a post-growth of grains, causing more grains to migrate and coalesce [20]. Laser irradiation results in the development of lateral facets and the migration of the ZnO grains, as well as the production of the large punctures between the grains, which can be more effective for relaxing the residual compressive stress in the as-grown ZnO films [20]. Although the ZnO films with laser annealing appear to have a porous structure, their application in pyroelectric devices is beneficial as porous ZnO film increases the absorption of heat radiation. Moreover, annealing in a nitrogen atmosphere suppresses the re-evaporation of oxygen and, hence, the non-radiative defects. Therefore, the properties of ZnO films are improved by annealing and the density of defects in ZnO films is decreased [21].

A three-dimensional pattern has been presented and etched on the responsive element of LiTaO_3_ by using lateral temperature gradients produced on the sidewalls of the responsive element to enhance the pyroelectric response [7]. In the present study, a two-dimensional finite element model was generated by the commercial multi-physics software COMSOL MULTIPHYSICS^®^ 3.5 to explore the temperature variation rate in ZnO pyroelectric devices with both comb-like and fully covered electrodes. Material parameters of the ZnO film, the substrate and electrodes are listed in Table 4.

**Figure 7.**
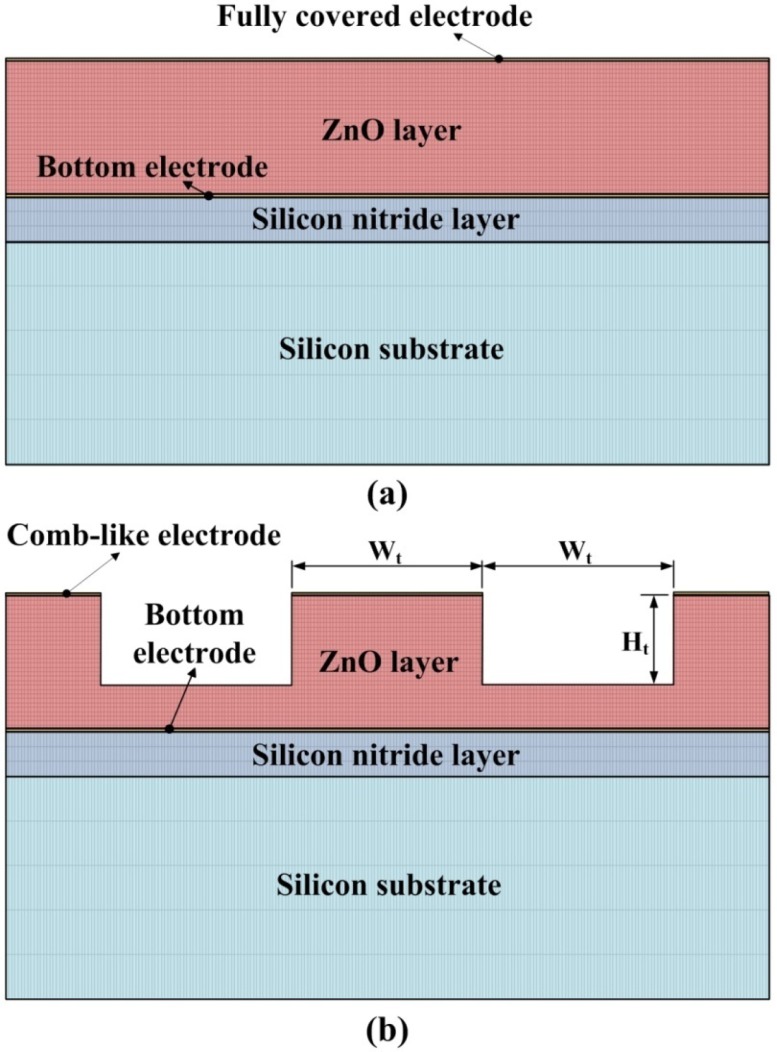
Finite element model for the ZnO pyroelectric devices: (**a**) fully covered electrode; (**b**) comb-like electrode.

All parameters were assumed to be isotropic. The models were meshed by regular mesh, as shown in Figure 7. Fitting the structure of patterned devices, W_t_ is defined as the electrode width and set at 500 μm. H_t_ is defined as the trench depth and set at 2 μm. The incident irradiation power applied to the top side of the ZnO pyroelectric devices was approximately 1.228 × 10^−12^ W/μm^2^[22]. Although the vertical sidewalls of the trenches had a high potential to absorb the incidental irradiation power, there was no irradiation power applied to make a worst-case scenario for probing the temperature fields and predicting the efficiency of ZnO pyroelectric devices. Moreover, thermal isolation was applied to the rear side of the ZnO pyroelectric devices, and the two lateral sides were symmetric in order to serve as boundary conditions.

**Figure 8.**
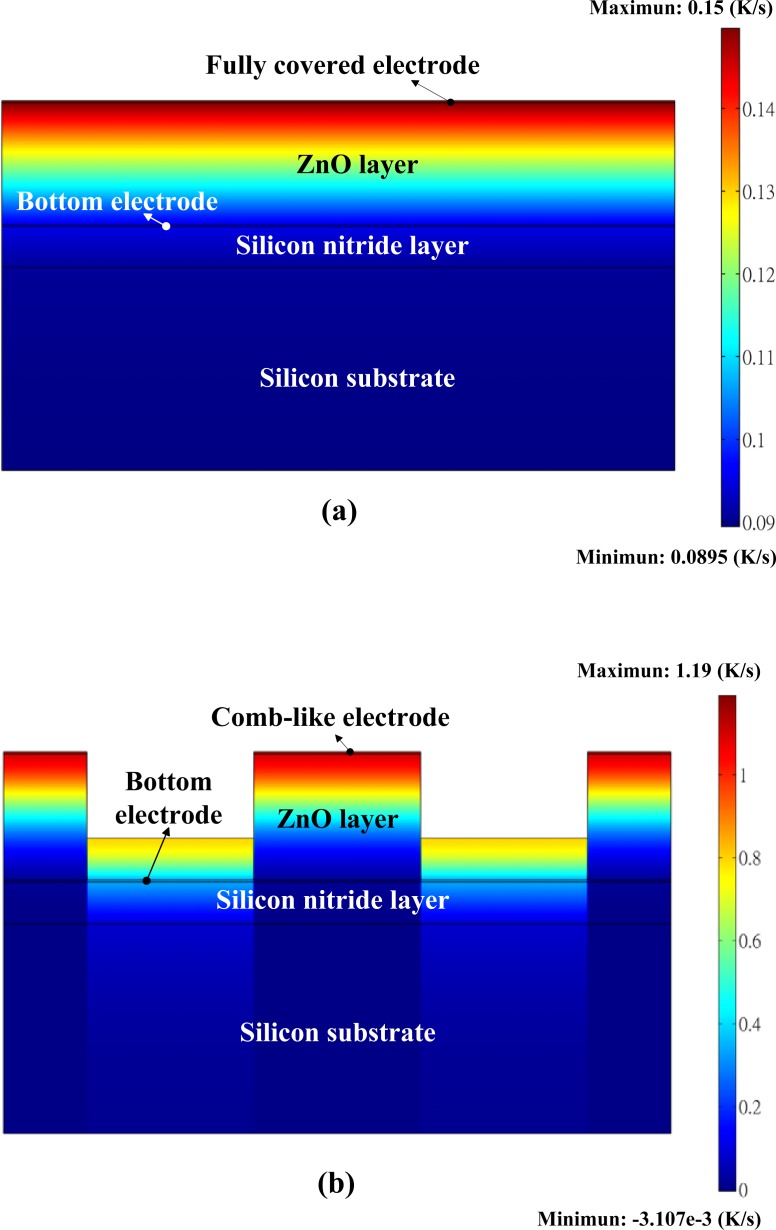
Transient temperature variation field in the ZnO pyroelectric sensors: (**a**) fully covered electrode; (**b**) comb-like electrode.

Figure 8(a) shows the transient temperature variation field at the time of the maximum peak at the bottom point of the ZnO film when the fully covered electrode was used to fabricate the ZnO pyroelectric devices. The temperature variation rate in the ZnO film increased gradually toward the top electrode as a result of the incidental radiation power applied to the top electrode. Figure 8(b) shows the transient temperature variation field at the time of the maximum peak at the bottom point of the ZnO film when the comb-like electrode with an electrode width of 500 μm and a trench depth of 2 μm was used to fabricate the ZnO pyroelectric devices. Because the incidental radiation power was applied on the top side of the ZnO pyroelectric devices, the temperature variation rates at the top side of the ZnO film under both the comb-like and the fully covered electrodes were no different. Obviously, the temperature variation rate at the bottom side of the ZnO film was improved about 32% by lateral temperature gradients. The three-dimensional ZnO film could bring lateral temperature gradients on the sidewalls of the ZnO layer and so increase the temperature variation rate of the responsive element.

**Figure 9.**
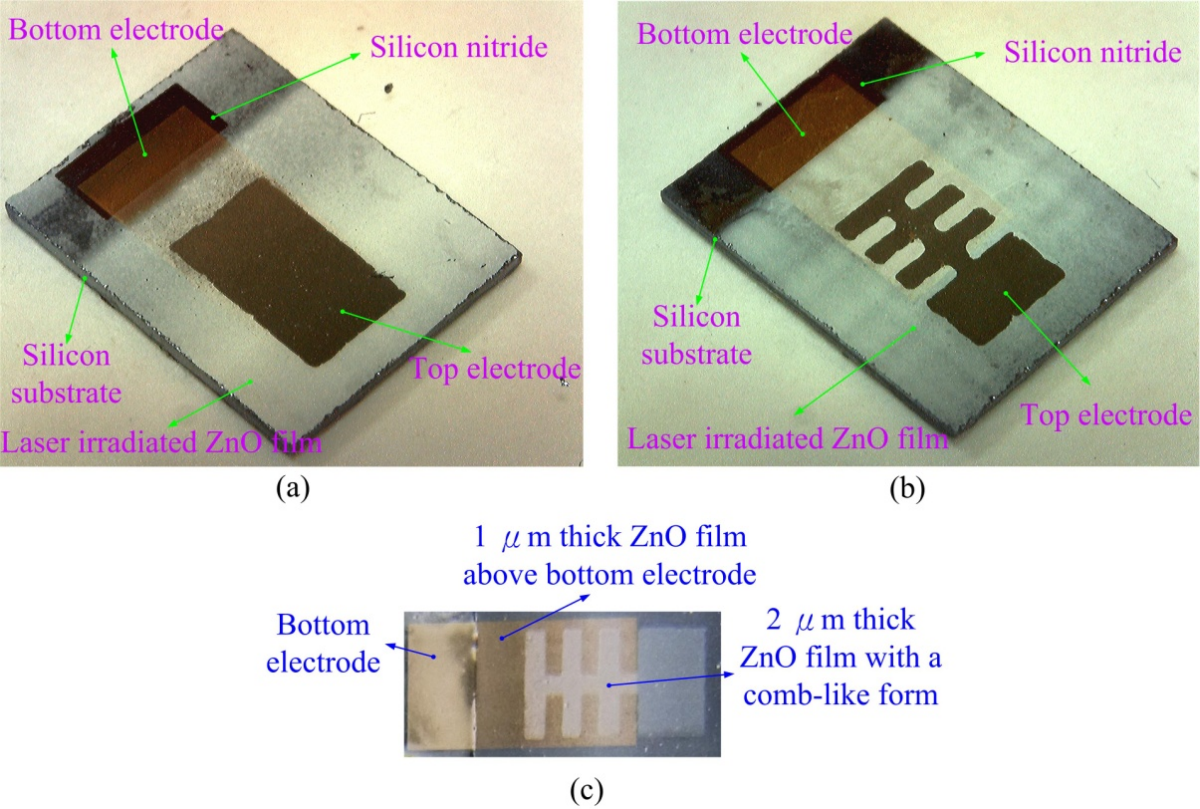
Fabricated ZnO pyroelectric devices: (**a**) the fully covered electrode with the single ZnO layer; (**b**) the comb-like electrode with the three-dimensional ZnO film; (**c**) the patterned device before top electrode deposited.

When ZnO is subjected to temperature variation, its internal polarization produces an electrical field that induces a voltage response between the top and bottom electrodes. The response is proportional to the temperature variation rate in the ZnO layer, as no temperature variation in the ZnO layer results in no internal polarization change and, thus, no response voltage. Therefore, the IR laser beam was chopped and molded as a square wave with a modulated frequency to obtain a temperature variation in the ZnO layer by a programmable function generator. In the present study, the AD method was used to fabricate a multilayer ZnO pyroelectric device with a comb-like top electrode and three-dimensional ZnO film, as compared to a fully covered top electrode and a single ZnO layer. The top electrode area of the fully covered type was about 14.4 mm^2^, which was larger than that of the comb-like top electrode at about 10.6 mm^2^. The single ZnO layer possessed a thickness of 3 μm, which was the same as the three-dimensional ZnO film. The fabricated ZnO pyroelectric device with the fully covered electrode and the single ZnO layer, compared to that with the comb-like electrode and three-dimensional ZnO film, fabricated through ZnO film deposited by AD, are shown in Figure 9.

**Figure 10.**
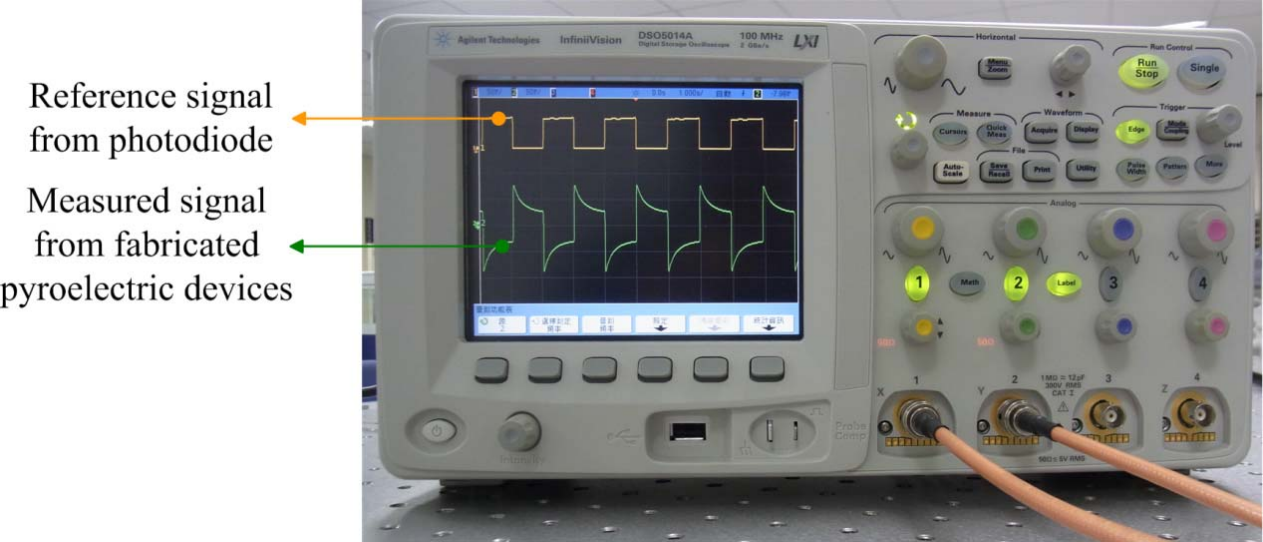
Voltage responsivity waveform of the present ZnO pyroelectric devices, compared to that of the reference signal of the photodiode on the oscilloscope.

Figure 10 shows the waveform of the voltage response of the present ZnO pyroelectric device compared with that of the reference signal of the photodiode on the oscilloscope. The signal of the fabricated ZnO pyroelectric device presented a harmony with the reference signal of the photodiode. Because pyroelectric devices start applying the temperature variation rate, the waveform of the voltage response rapidly rises to a maximum value and then slowly decreases due to the sustained radiation source applied. Therefore, increasing the heat absorption of porous film is helpful in enhancing the voltage response of pyroelectric devices.

**Figure 11.**
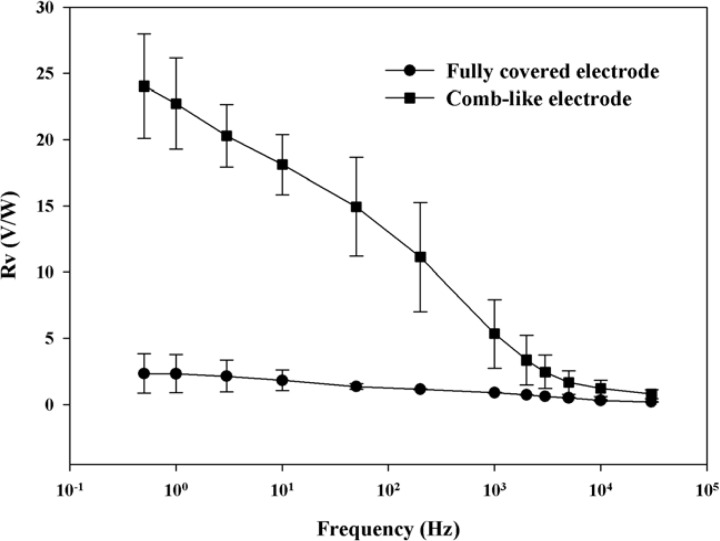
Voltage responsivity of the fabricated ZnO pyroelectric sensors with the comb-like electrode, compared to the fully covered electrode.

Figure 11 shows the voltage response (*Rv*) of the present ZnO pyroelectric devices with ZnO film deposited by AD and ameliorated by laser annealing.

There were six samples for each electrode layout. *Rv* is defined as the ratio of the output voltage of the device to the input power of the incident heat source. The voltage response of the pyroelectric sensor was as expected, in that it decreased as the modulated frequency increased. Moreover, as the frequency went beyond 1,000 Hz, the voltage response seriously decreased with increasing modulated frequency. This was due to the fact that the response time of the sensor could not catch the variation speed of the incident heat source. Moreover, the voltage response still had a good performance at the period of 33 μs. The ZnO pyroelectric device with the comb-like electrode possessed a voltage response about 9 times greater than that with the fully covered electrode at a low frequency. At a high frequency of about 3,000 Hz (33 μs), the ZnO pyroelectric device with the comb-like electrode possessed a voltage response about four times greater than that with the fully covered electrode. Although the fully covered electrode possessed a larger electrode area than the comb-like electrode, the radiation absorption of the ZnO layer was seriously obstructed by the fully covered top electrode. Therefore, increasing only the top electrode area without consideration of the electrode layout and three-dimensional ZnO film could not effectively improve the response of pyroelectric devices. Hence, AD did indeed accelerate the deposition process of ZnO films for applications in pyroelectric devices, as well as reduce the manufacturing cost for the fabrication of microdevices. Moreover, the porous ZnO films with the three-dimensional pattern fabricated by AD and integrated with the comb-like top electrode improved the voltage response of pyroelectric devices.

## 4. Conclusions

In this study, AD was successfully used to accelerate the application of ZnO films in pyroelectric devices. A three-dimensional ZnO film fabricated by AD with the shadow mask method, integrated with a comb-like top electrode, enhanced the voltage response of ZnO pyroelectric devices. At a high frequency of about 3,000 Hz (33 μs), the ZnO pyroelectric device with the comb-like electrode possessed a voltage response about four times greater than that with the fully covered electrode. The three-dimensional ZnO film did indeed induce lateral temperature gradients on the sidewalls of the ZnO layer, thereby increasing the temperature variation rate of the responsive element, enhancing the voltage response and reducing the response time of ZnO pyroelectric devices.

## Figures and Tables

**Table 1.** Properties of ZnO powder.

**Item**	**Data**
Appearance	White powder
Density	5.61 g/cm^3^
Specific surface area	17 m^2^/g
Particle form	Sheet
Particle size	300 nm (Diameter) × 20 nm (Thickness)

**Table 2.** Process parameters for ZnO film deposited by AD method.

**Item**	**Data**
Starting powder	ZnO
Substrate	Silicon
Pressure difference between deposition and aerosol chambers	140 (Torr)
Carrier gas	Nitrogen
Consumption of carrier gas	3 (L/min)
Orifice size of nozzle	0.4 × 10 (mm × mm)
Substrate temperature	25 (°C)
Deposition area	70 × 70 (mm × mm)
Distance between nozzle and substrate	5 (mm)
Scanning rate	10 (mm/s)
Deposition rate	8.2 (nm/s)

**Table 3.** Process parameters for ZnO film treated by laser annealing.

**Item**	**Data**
Laser type	Continuous-wave CO_2_ laser
Laser power	25 (W)
Movement velocity of laser head	1.4 (mm/s)
Distance between substrates and laser head	40 (mm)
Ambient gas	Nitrogen
Flow rate of ambient gas	3 (L/min)
Laser spot	2 mm (diameter)

**Table 4.** Material parameters used for finite element analysis.

**Material**	**Thermal conductivity (Wm^−1^·K^−1^)**	**Specific heat (Jg^−1^·K^−1^)**	**Density (g·cm^−3^)**	**Thickness (μm)**
**Silicon substrate**	163	0.703	2.330	5
**Silicon nitride**	20	0.700	3.100	1
**Top and bottom electrodes**	317	0.129	19.300	0.1
**Zinc oxide**	6	0.125	5.676	3
